# Chemical Composition and Quality Characteristics of Meat in Three One-Humped Camel (*Camelus dromedarius*) Breeds as Affected by Muscle Type and Post-Mortem Storage Period

**DOI:** 10.3390/ani9100834

**Published:** 2019-10-20

**Authors:** Gamaleldin M. Suliman, Abdullah N. Alowaimer, Elsayed O.S. Hussein, Hatem S. Ali, Sameh A. Abdelnour, Mohamed E. Abd El-Hack, Ayman A. Swelum

**Affiliations:** 1 Department of Animal Production, College of Food and Agricultural Sciences, King Saud University, P.O Box 2460, Riyadh 11451, Saudi Arabia; 2 Department of Meat Production, Faculty of Animal Production, University of Khartoum, Khartoum 11111, Sudan; 3 Food Science and Nutrition Department, College of Food and Agricultural Sciences, King Saud University, Riyadh 11451, Saudi Arabia; 4 Food Technology Department, National Research Center, Dokki, Cairo Governorate 12622, Egypt; 5 Department of Animal Production, Faculty of Agriculture, Zagazig University, Zagazig 44511, Egypt; 6 Department of Poultry, Faculty of Agriculture, Zagazig University, Zagazig 44511, Egypt; 7 Department of Theriogenology, Faculty of Veterinary Medicine, Zagazig University, Zagazig 44511, Egypt

**Keywords:** chemical composition, meat quality and sensory properties, muscles, aging, one-humped camel

## Abstract

**Simple Summary:**

The present study aimed to investigate the influences of muscle type and postmortem storage period on meat chemical composition and quality attributes of three breeds of camels, including Baladi Saudi, Pakistani, and Somali. Baladi Saudi and Pakistani had better carcass characteristics than Somali. In addition, the *longissimus lumborum* muscle had better meat quality and the storage process can enhance quality features of meats that have relatively small amounts of connective tissue and that have not cold-shortened.

**Abstract:**

The influence of muscle type and postmortem storage period on meat chemical composition and quality attributes of three breeds of camels (Baladi Saudi, Pakistani, and Somali) were investigated in this study. Crude fat and ash content were significantly higher in the Pakistani than in the Baladi Saudi and Somali breeds, except for higher moisture content observed in the Somali breed. The longissimus lumborum (LL) and semimembranosus (SM) muscles had a greater crude protein than the biceps femoris (BF) muscle. Storage period exhibited a significant reduction in pH values and improvement in color components of meat. The Somali breed produced higher cooking loss % and shear force, with a lower water holding capacity than the Baladi Saudi and Pakistani breeds. The LL muscle had better cooking loss %, water holding capacity, and shear force, whereas storage period (7 days) exhibited a significant reduction in the myofibrillar fragmentation index. Baladi Saudi and Pakistani breeds and LL muscle samples presented better meat sensory attributes, while storage period had no significant influence on the overall sensory characters of meat. In conclusion, there were significant differences between the chemical and structural characteristics of the LL, BF, and SM muscle samples among the three breeds of camel. Baladi Saudi and Pakistani had better meat quality traits than the Somali breed. In addition, LL muscles had better nutritional values and meat quality parameters than BF and SM muscles. Improvement in meat quality attributes were achieved with the storage process of 7 days. It is observed that, the Saudi Baladi camels have a merit of low fat content over both Somali and Pakistani camel breeds. It is also concluded that no significant effects were observed between the treatments as a result of storage when sensory attributes were considered. Moreover, breed, muscle and storage period were interacted significantly only with regard to lightness color space and shear force. This is useful knowledge for the meat industry for optimizing processing and storage procedures for various camel muscles.

## 1. Introduction

The Arabian dromedary camel or the one-humped camel (*Camelus dromedarius*) is common in the dryland and hot arid areas of the Middle East, Asia, and Africa, while the Bactrian two-humped camel (*Camelus bactrianus*) is distributed in central Asia, China, Kazakhstan, and Russia [1]. *C. dromedarius* is exceptionally well-adapted to drought, is heat tolerant, and is able to survive and produce high quality animal protein under extremely harsh environmental conditions in comparison to other domestic animals [2,3,4]. The population of dromedary camels is increasing worldwide because of the unique features of camels, including an exceptional ability to survive and thrive under adverse climatic conditions such as high ambient temperatures, low rainfall, and feed scarcity. Moreover, considering the similarity of the properties of camel meat to cattle meat for numerous quality traits, the increasing acceptability of camel meat globally, and their therapeutics uses, there are huge opportunities for technological development of camel meat traits [5,6]. Therefore, the role of the camel as a meat producer is gaining more attention owing to the versatile role it plays rather than the previous role as a symbol of social prestige, which has since greatly diminished [2]. As was reported by several authors [3,4], the meat quality of camels varies significantly according to breed, sex, age, housing system, diet, pre-slaughter handling, and post-slaughter processing. The genetic material has a significant effect on the chemical composition and meat quality attributes. The technological enhancement of camel quality traits, such as improving cooking loss, color, flavor, tenderness, juiciness, and acceptability, increasing MFI%, and reducing shear force could lead to the development of expanded markets for camel meat not only in the Middle East and North Africa but also globally. Considering this, there is a dearth of information on the meat quality attributes of these camel breeds, and more research is required to reduce the effects of genetics, muscle type, and storage period on the quality of meat.

Somali camel breed has largest body size (average 550 kg), average milk yield of 4.5 L, heavy feeder, does not fit in hilly terrain, largely creamish in coat color and genetically distinct. Pakistani camel breed characterizes by smaller body size than Somali (average 400 kg), highest milk yielder (average 10 L), gross feeder, yet to be tested on terrain, mainly grey in color, genetically distinct. Baladi Saudi breed has medium body size (average 500 kg), average milk yield of 8 L which can reach to 16.6 L milk per day under improved management system, heavy feeder, does not fit in hilly areas, has different coat colors and genetically distinct.

Camels have multifunctional uses not only for meat, milk, and transportation proposes, but their uses have also expanded to more value-added products. Camel milk is well-known for its medicinal benefits that have been extensively exploited for human health; therefore, since the last few decades, the milk is processed into industrial dairy products such as yoghurt and ice cream [7]. In addition, meat is processed into food items such as sausages, shawarma, burgers, and patties and global camel meat production has increased by 100% between 1979 and 2009, representing about 1.3% of the world’s meat production [8]. In recent years, there has been an increase in the demand for camel meat as an alternative food characterized by several unique features. These include high protein content, low fats, and relatively high proportion of polyunsaturated fatty acids, in addition to favorable meat quality characteristics [9]. This emerging meat source provides an alternate available food source under harsh environmental conditions for the growing human population [10].

Camel meat contains 76–78% moisture, 19% protein, 2.9–3% fat, and 1.2% ash with a dressing percentage of about 55–70% and is thus considered a good source of nutrients [11,12,13,14], while the meat characteristics vary depending on several factors including age, sex, feeding programs, weight, and cut. Camel meat is distinctively lean, with a lower fat content, lower cholesterol, and higher essential amino acids and polyunsaturated fatty acids than other meat-producing animals like cattle and sheep. These features increase the demand of camel meat and camel-based products [9,10,12]. Conventionally, camel meat is mostly produced from older animals (more than 7 years of age) after completing a career in transportation, racing, and milk production. Therefore, the common opinion among consumers is that camel meat is tough and firm [15,16].

Longissimus lumborum (LL), biceps femoris (BF), and semimembranosus (SM) muscles are of special importance to meat producers and consumers. Biceps femoris (BF) muscles have a higher content of fats, minerals and moisture than longissimus lumborum (LL), muscles and may be similar in the chemical composition with semimembranosus (SM) muscles in camel meat [3]. Hence, from quality and economical point of view, these muscles were investigated in our study.

Despite this, the quality of meat from younger camels and the relationship of age to the structural characteristics of camel muscle or meat have received little attention. Hence, the objectives of this study were to assess chemical composition, physicochemical parameters (pH, color, cooking loss, water holding capacity), structural characteristics (myofibril fragmentation index, texture profile analysis, shear force), and sensory parameters of meat from young (1.5 and 2 years of age) camels of three Saudi Arabian breeds (Baladi Saudi, Pakistani, and Somali). The effect of muscle type and the storage period was considered.

## 2. Material and Methods

### 2.1. Animals and Ethics Approval

Twenty-four camels from three breeds, Baladi Saudi, Pakistani, and Somali (8 camels per breed), were selected for comparison. Their average live weight and age were 225 ± 5 kg and 1.5–2 years old, respectively. All animals were kept under the same environmental condition and received the same management. They were housed in partially shaded pens supplied with individual feeders and water troughs. They fed a commercial concentrate mixture supplemented with alfalfa hay and free access to water and salt-lick blocks as a source of micronutrients. The animals were slaughtered according to the food industry-approved halal food quality certified protocol guided by the legislations of Islam. Although however, the study was conducted following the recommendations of work on live animals set by The Scientific Research Ethics Committee (SREC), College of Food and Agricultural Sciences, King Saud University, Saudi Arabia.

### 2.2. Slaughtering and Treatment of Samples

Eight animals from each breed were slaughtered following Halal procedure that came in accordance with the Islamic legislations [9]. In this method of slaughter, the camels were bled without stunning. Then, immediately the main blood vessels; jugular veins and carotid arteries were cut with a sharp knife. After exsanguination, the carcasses were split into two equal halves along the vertebrate column, then three different muscles, including longissimus lumborum (LL), biceps femoris (BF), and semimembranosus (SM) muscles were immediately collected from the left side of the animal carcass, separated from the bones, and transferred at 4 °C to the Meat Production and Quality Laboratory at the Department of Animal Production, College of Food and Agricultural Sciences, at the King Saud University (Riyadh), Saudi Arabia. The samples of the LL muscle (loin cut) were dissected from the last rib of the left side of each carcass. Five samples were collected from each muscle for further analysis. The weight of each sample varied according to the type analysis and ranged from (2–400 g). These samples were allocated into three equal portions with the fat removed from each muscle. The first represented the control group and was labelled as Day 0 (immediately after slaughtered); then, each sample was further cut into nine pieces and the following tests were performed within 3 h from sampling: Moisture + ash, ether extract, crude protein, sensory evaluation, cooking loss + shear force, myofibril fragmentation index, and water-holding capacity. The second and third portions were labelled as Day 3 and Day 7 representing 3 and 7 days of storage period, respectively. They were subjected to wet storage period where they were stored in sealed, oxygen-impermeable vacuum bags and kept at refrigerated temperature (1 °C) for the relative storage period. At the end of storage period, the samples were stored frozen at −20 °C until further analysis.

### 2.3. Chemical Composition

Two hundred grams of each meat sample were placed in plastic containers, frozen, and then dried in a thermo freeze dryer (Modulyol-230, Milford, UK) under 100 mbar pressure at −50 °C. The frozen dry samples were ground to a homogeneous mixture in a mixer for chemical analyses. At 48 h postmortem, the LL, BF, and SM muscles in each breed were used to assess moisture, crude protein, crude fat, and ash according to the procedure described by the AOAC [17]. In brief, moisture was assessed using the weight of a 50 g meat sample before and after drying in a thermo freeze dryer for 5 d. Protein content was evaluated (method 976.95) using Kjeltec 2300 nitrogen/protein analyzer (FOSS Analytical 69, Slangerupgade DK-3400 Hilleroed, Denmark), and the Soxhlet extraction method was used for determination of fat content (method 920.39). Finally, ash content was evaluated by ashing samples in a muffle furnace at 500 °C for 24 h (method 942.05).

### 2.4. Muscle pH, Color Values, Chroma and Hue Angle Value

Both pH and color space values were measure 24 h after slaughtering and storage of samples at 2 °C. The pH was determined using a pH meter equipped with a penetrating glass electrode (Model pH 211, Hanna Instruments, Woonsocket, Rhode Island, RI, USA). Three readings were taken and the mean value was calculated. The CIELAB Color System 1976 [18] color space values (a* for redness, L* for lightness, and b* for yellowness) were assessed using a colorimeter (Konica Minolta, CR-400- Japan; Measuring aperture: 8 mm; Illuminant: CIE D65; Observer angle: CIE 2° Standard Observer). Reflectance measurements were performed after the samples had oxygenated in air for at least 30 min by which time measurements were stable. Three readings were taken on the muscle surface, then and a mean value was processed. Chroma was determined using the values of a* and b* according to the following equation: chroma = (a*2 + b*2)1/2 and hue (H°) = /tan-1 (b*/a*) using the methods described by Mancini and Hunt [19] and Little [20].

### 2.5. Water Holding Capacity (WHC)

Water-holding capacity was performed following the methodology described by Wilhelm et al. [21]. Two grams of each meat sample were tested for the WHC and expressed as the percentage of expelled water using the filter paper method as the total wetted area less than meat film area (cm^2^) relative to sample weight (g).

### 2.6. Myofibril Fragmentation Index (MFI)

The MFI of the meat samples from the three breeds was obtained using the method outlined by Culler et al. [22]. In brief, 4 g of the muscle sample was minced using a scissor, then homogenized in a mixer with 40 mL of cold (2 °C) MFI buffer (100 mM KCl, 20 mM potassium phosphate, 1 mM EGTA, 1 mM MgCl_2_, 1 mM NaN_3_). Thereafter, several washes were performed, and then the absorbance of the resultant 0.5 mg/mL solution was read at 540 nm using spectrophotometer (HACH DR/3000 Spectrophotometer, USA). The MFI of each sample was calculated by multiplying the absorbance at 540 nm by 200.

### 2.7. Cooking Loss (CL)

The frozen steaks were thawed overnight at 4 °C. At the time of measurement, a 2.5-cm-thick muscle sample (about 400 g) was taken to perform the test. The sample was placed in an electric commercial stainless-steel grilling oven and cooked at 200 °C to an internal temperature of 70 °C. The internal temperature was observed by inserting a thermocouple probe (Ecoscan Temp JKT, Eutech Instruments, Pte Ltd., Keppel Bay, HarbourFront, Singapore) into the center of each steak. After cooking, the steaks were cooled at room temperature (18 °C), surface dried with filter paper, and reweighed using an analytical balance (Mettler AE1 00-0.001-Mettler-Toledo, LLC 1900 Polaris Parkway Columbus, OH 43240, USA). Cooking losses were calculated from differences in raw and cooked weight as: Cooking loss = (weight of raw sample − weight of cooked sample) × 100/weight of raw sample [9].

### 2.8. Shear Force (SF) and Texture Profile Analysis (TPA)

The shear force for the samples was assessed following the procedure detailed by Wheeler et al. [23]. The samples used for measuring cooking loss were reused to assess shear force. Three round cores (1.27 cm in diameter) were removed from each muscle sample parallel to the longitudinal orientation of the muscle fibers using a manual coring device. The shear force was obtained as the maximum force (N) perpendicular to the fibers using a TA.HD Texture Analyzer (Stable Micro Systems, Surrey, UK) outfitted with a Warner-Bratzler attachment. The crosshead speed was set at 200 mm/min. The same methodology of samples preparation and cooking used for SF was applied for texture profile analysis (TPA) components determination. The TPA was conducted using the Texture Analyzer (TA.HD, Stable Micro Systems, Surrey, UK) fitted with a compression-plate attachment. Each sample underwent two cycles of 80% compression. The components determined were hardness, cohesiveness, springiness, and chewiness.

### 2.9. Assessment of Sensory Parameters

The cooked meat (about 400 g) that had been prepared for shear force measurements was assessed for tenderness; the assessment was carried out in a room at ambient temperature (18 °C) and in daylight. The quality of cooked meat was evaluated according to an 8-point scale as described by American Meat Science Association (AMSA) [24]. A team of eight semi-trained panelists assessed color, flavor, tenderness, juiciness, and acceptability (desirability). Each descriptor was scored from 1 (minimum) to 8 (maximum). The assessors received three samples each 2 cm in size (about 50 g) that had been cooked in disposable plastic containers with lids in a dry oven at 120 °C for 30 min. The carry-over effect was avoided by encoding the sample names. The assessments were performed twice. After assessment of each sample, the panelists neutralized its taste by drinking unsweetened tea between tastings the samples. The sighted persons were given pens and evaluation sheets for systematic completion during testing.

### 2.10. Statistical Analysis

Comparisons among groups were analyzed based on a factorial design using repeated measures analysis of variance (ANOVA), using SPSS^®^ software program version 21 (SPSS, Chicago, IL, USA) to evaluate the effect of breed, muscle type, storage period and their interactions [25,26]. A difference was considered significant at *p* < 0.05 level. Data were expressed as the mean± standard error.

## 3. Results

### 3.1. Effects on Chemical Composition

The effects of breeds (Baladi Saudi, Pakistani, and Somali) and type of muscle (LL, BF, and SM) on chemical composition (moisture, crude protein, crude fat, and ash) are shown in Table 1. Breed had a significant effect on crude fat and ash values (*p* < 0.0001 and *p* < 0.01, respectively) with the Pakistani camel samples having higher crude fat and ash than the Baladi Saudi and Somali samples. However, no significant differences were observed on crude protein percentage among breeds. In addition, significant differences were detected in crude protein, crude fiber and ash between muscle types. The LL muscle recorded the highest crude protein and crude fat values (21.92; *p* < 0.04 and 4.37%; *p* < 0.02, respectively) and had the lowest moisture values (72.77%) compared to BF and SM samples. LL samples had 20.05% and 21.05% higher amount of fat than BF and SM samples, respectively. BF samples had no significant differences in chemical composition parameters studied when compared with SM samples except for ash. The interaction between type of muscle and breed had no effect (*p* > 0.05), except for crude protein.

### 3.2. Effects on pH and Color Components

The effects of breed, type of muscle, and storage period and their interactions on pH and color components of camel meat are presented in Table 2. Breed and storage period had a significant effect on pH and color (except b* for breed and L* for storage period). The highest pH values (5.99) were recorded for the Pakistani breed than for other breeds (*p* < 0.01) which did not differ from each other. The Baladi Saudi and Pakistani breeds had higher L* values than the Somali breed, while a converse trend was observed in a* values with the Somali breed having the highest value. There were no significant effects of camel breed on chroma, b*/a* ratio, and hue angle traits. Significant effects of types of muscle were detected on color coordinates (except for L*), chroma, b*/a* ratio, and hue angle. The LL muscle samples exhibited significantly higher values of a* (15.61) and b* (9.77) coordinates, chroma (18.62), b*/a* ratio (0.66%), and hue angle (31.93) than BF and SM muscle samples. 

However, SM muscles samples recorded higher values for a*, b*, chroma, b*/a* ratio and hue angle, no significant differences were found between BF and SM samples for these traits.

As expected, storage period treatments had significant (*p* < 0.0001) effect on pH, color components (except the L* coordinate) and *, Chroma, b*/a* ratio and hue angle of camel meat.

Only pH values tended to be lower in association with the increase in the time of storage period (7 d); however, significantly higher values of b* coordinate, chroma, b*/a* ratio and hue angle were observed in camel meat aged 7 days. For the interaction between breed and time of storage period exhibited significant effect on pH and color components of camel meat. In addition, the interaction between type of muscle and storage period showed significant effect (*p* = 0.02) for pH and also, the interaction among breed, type of muscle and storage period was exhibited significant influence (*p* = 0.07) for L* values.

### 3.3. Effects on CL, WHC, MFI, SF, and TPA

Results of the effects of breed, type of muscles, and storage period and their interaction with meat attributes (CL, WHC, MFI, SF, and TPA) are shown in Table 3. Meat quality attributes including MFI and TPA (hardness, springiness, and chewiness; except Cohesiveness for type of muscle effect) were not affected by breed, muscle differences, and storage period treatments (*p* < 0.05). It was evident that the Somali breed had a higher CL (34.28%) and SF (46.58 N) (*p* < 0.01, *p* < 0.0001, respectively) than that of other breeds, whereas the Pakistani breed recorded the higher value (*p* < 0.05) for WHC. Both of the two breeds Baladi Saudi and Pakistani did not exhibited significant differences regarding the values of SF. Regarding muscle type, BF and SM had a higher CL and WHC than LL, whereas there were no differences in LL and SM for WHC (*p* < 0.05). SF showed significant differences in relation to the type of camel muscles (*p* < 0.0001). The highest values for SF (40.31 N) were observed in BF when compared with SM (35.60 N) and LL (31.28 N) muscles. Cohesiveness exhibited significant differences according the type of muscle, where the highest values for Cohesiveness (0.52) were observed in BF muscles than LL and SM muscles. Moreover, regarding the effects of storage period treatment, MFI values tended to get lower with storage period and the lowest value was observed at 7 days of storage period (*p* < 0.01). Additionally, no significant effects were observed giving the effect of type muscle on CL, WHC, SK and TPA attributes. The breed × storage period and breed × muscle × storage period effects were significant for SF; in contrast, the other interaction effects were non-significant for all traits.

### 3.4. Effects on Sensory Evaluation Parameters

The effects of breed, muscle type, storage period, and their interaction on muscle color, flavor, tenderness, juiciness, and acceptability are presented in Table 4. All sensory evaluation parameters showed significant differences (except for flavor) by breed. In addition, the muscle type had significant influence on these meat quality attributes. The Baladi Saudi and Pakistani breeds had significantly higher tenderness (*p* < 0.0001), juiciness (*p* < 0.0001) and acceptability (*p* < 0.01) values than the Somali breed. No significant differences were indicated for color among Somali and Pakistan breeds; however, the lowest values were observed in the Baladi Saudi breed (3.15; *p* < 0.01) demonstrating that the effects of muscle types were clear on sensory evaluation parameters. Flavor, tenderness, juiciness, and acceptability were higher in LL muscle than in other muscle samples (*p* < 0.03, *p* < 0.0001, *p* < 0.01, *p* < 0.01, respectively). Conversely, it was observed that LL samples had a significantly lower color value (3.2) than SM and BF muscles (3.65 and 3.54, respectively, *p* < 0.01). No significant effects were observed with storage period treatment on color, flavor, tenderness, juiciness, and acceptability values and the interaction effects were also non-significant for all studied traits.

## 4. Discussion

This study illustrates the effects of breed, muscle type, storage period, and their interaction on pH, color components, and meat quality of three breeds of camels (Baladi Saudi, Pakistani, and Somali).

Results of many previous studies indicated that the chemical composition of camel meat is mostly similar to the meat from other species where converse associations were recorded between the moisture, and protein and fat contents of the meat [3,9,10,12,27]. Meat composition is considered a significant indicator of meat functionality; protein and fats are essential constituents reflecting the quality value of meat, whereas moisture content plays a central role in eating and keeping qualities of camel meat [2,3,12]. The Somali and Baladi Saudi breeds had lower moisture content and higher ash content; however, no significant differences were observed between breeds.

Concerning the muscle type, our results showed higher protein content in LL (21.92%) and SM (21.11%) than BF (20.45%). The normal range for protein content in camel meat is approximately 17–23.7% as reported by Kadim et al. [10]. In this study, a small difference between various muscles for protein content was observed; however, this variation was significant and the results are consistent with previous studies [16,28,29]. Moreover, consistent with our findings, the range of moisture content of BF muscle samples and SM muscle samples was higher than that from LL muscles owing to the higher protein content in the LL muscle. Differences in protein and moisture content by muscle type in camel meat were reported by Kadim et al. [10] and Raiymbek et al. [29]; higher moisture % was observed in the biceps femoris (74.3–78.5%) and triceps brachii (77.7–78.4%) muscles than in the longissimus dorsi muscle (72.1–73.8%). The authors suggested that this difference may be related to the higher fat content in the longissimus thoracis muscle. There were no significant differences between muscle type for fat and ash content in camel meat in the present study. According to previous studies, it has been reported that the content of fat in camel meat ranges from 1.1 to 10.6%. Differences in the fat content in various camel muscles were reported [12,16,26,27,29,30,31]. Gheisari et al. [27] reported lower fat content in BF than in LL muscles. It is generally accepted that camel meat has comparatively lower fat content than meat of other species such as goat, lamb, and cattle [12,26]. These results indicated that LL muscles can be considered one of the best choices for quality meat in comparison to other muscles in the camel and SM muscles have an almost similar chemical composition.

### 4.1. Effects on pH and Color Components

The eventual pH in muscles is a result of lactic acid accruement via glycolysis that affects meat quality attributes [32,33]. Regarding pH in muscles, breed had a significant effect. The Pakistani breed produced higher pH values (5.99) in comparison to the Baladi Saudi and Somali breeds (5.87 and 5.90, respectively). Moreover, after the storage period treatment, muscle pH showed significantly higher values. In contrast, the muscle type had no effect on the pH values. The muscle pH values are affected by several complex factors such pre-slaughter handling, postmortem treatment, glycogen storage, and muscle physiology. The reduction in pH value after slaughtering mostly shows desirable effects on meat quality, preservation and preservation period, and the microbial activity, all of which influence consumer acceptance of camel meat. Low muscle glycogen, stores at slaughter, prevents the development of a desirable pH postmortem [34]. Our results were to some extent consistent with those of Babiker and Yousif [35] and Kadim et al. [10,16,30,31] who reported that the ultimate pH of dromedary camel meat ranges between 5.5 and 6.6. In fact, postmortem pH reduction in camel meat is significantly slower than that in beef [36]. It was therefore concluded that pH values reported in this study were consistent with other published results. However, the muscle type had no effect on pH value. Similarly, Suliman et al. [37] reported the non-significant effects of breed and muscles type (longissimus thoracis and biceps femoris) on the ultimate pH value. In this respect, Kadim et al. [16] suggested that the age of the camel is one of the main factors affecting pH value, where older camels tend to produce lower pH values than younger camels, because of high levels of glycogen content in the young camels. It has been proven that the process of aging is required to develop the attributes of good quality meat. Aging is a process that causes an enhancement in color, tenderness, pH, MFI, flavor, and texture over time and includes specific degradation of structural proteins [38]. It has been reported that storage period for 7 days at 2–3 °C enhanced attributes of camel meat quality [10]. The aging process had a significant effect on pH and color components of camel meat. Color components of meat camel affected by several factors; age, breed, sex, nutritional status, muscle and the period of aging [6,10,12]. We investigated the effects of breed, the type of muscle and storage period on the color components, and observed that these factors affected significantly on the color components. In accordance with our findings, it has been reported that the alteration in pH values and temperature of meat probably considered two of the main reliable issues can affecting the meat attributes with respect meat color [19]. These results were similar to those reported for the muscle of the one humped camel by Kadim et al. [10,16], Suliman et al. [37], and Al-Owaimer et al. [9]. This indicates that aging may be one of the postmortem management strategies that could be adopted in the camel meat industry to improve camel meat tenderness. Muscle type had a significant effect on a* and b*, chroma, a*/ b* ratio, and hue angle values.

### 4.2. Effects on CL, WHC, MFI, SF, and TPA

Cooking loss is one of the key features of meat quality that has a close association with WHC. Meat WHC is chiefly triggered by the immobilization of water within the myofibrillar meat tissues. Therefore, applying pressure can cause a change in water from the intercellular to the extracellular matrix and then onto the meat surface as a result of structural changes at the level of the myofilaments or sarcomeres [10]. Several factors affecting the CL and WHC of meat such as meat minerals, fats, protein, and water content have been established. It was reported that old camels had lower water holding capacity values than young animals [1,16]. Similarly, the study conducted by Shehata [39] revealed that young camels (10–12 months old) had a higher cooking loss than older animals. In our study, the meat of the Somali breed produced significantly higher cooking loss than both the Baladi Saudi and Pakistani breeds. Longissimus lumborum of camel meat had significant lower CL and WHC values than other muscle types. These results were consistent with previous studies [30,31]. The aging treatment of different periods had no effect on the majority of the meat quality attributes (except for MFI). The nutritional value of meat is concentrated or improved by the meat cooking process. Cooking loss is an important factor because it is likely to alter the amount of nutrients in the meat once it is cooked [27]. The TPA values of camel meat exhibited non-significant differences affected by genotypes, muscle type, and aging. It is noteworthy that a significant difference between breeds was observed for shear force, where among the three breeds, the Somali breed was characterized by the highest value of SF (*p* < 0.0001). However, in contrast to our findings, Al-Owaimer et al. [9] found that the genotypes of camels had no significant influence on SF values. Shear force is an indicator of toughness, which is associated with total protein, total collagen, and insoluble collagen content in the meat [9]. Moreover, muscle type has a significant influence on shear force, where the highest values were observed in BF muscle and the lowest in LL muscle samples. The results of MFI values of the camel meat were not statistically different, except for the storage period effect. The MFI is a valuable index of the extent of myofibrillar protein degradation in post-slaughter camel meat [10,16,29,30,31,32,33,34,35,36,37,38,39,40]. Nagaraj et al. [41] suggested that the changes in the rates of myofibrillar protein fragmentation may clarify the changes in the rate of postmortem tenderization of meat.

Likewise, Silva et al. [42] confirmed that the MFI values in meat were significantly higher at an ultimate pH of 6.5 than at pH 5.7. Veiseth et al. [43] found a close relationship between the MFI and the tenderness of meat. The MFI of camels above 6 years of age was lower than that of 1–3 years of age (10, 27). In addition, some authors reported a strong relationship between physical disruptions of the myofibrils and the tenderness of camel meat [9,30]. The results of the effects of storage period treatment on color, flavor, tenderness, juiciness, and acceptability values were not significant (Table 4).

### 4.3. Effects on Sensory Evaluation Parameters

The science of sensory evaluation has undergone tremendous development in the last 25 years [24]. Meat color is one of the principal significant sensory features according to which consumers make judgments on meat quality. The effects of breed, muscle type and storage period had a significant influence on all sensory evaluation attributes in camel meat (Table 4). Consequently, the assessment of tenderness, juiciness, and acceptability of the Baladi Saudi and Pakistani breeds received higher values than the Somali breed (*p* < 0001, *p* < 0.0001, and *p* < 0.01, respectively). In addition, the LL muscle sample had higher significant values for flavor, tenderness, juiciness, and acceptability than BF and SM muscle samples. The present study recorded non-significant effects of storage period on sensory meat attributes with non-significant interaction between storage period × breed or muscle type. Tenderness is the most important organoleptic characteristic and is the predominant determinant quality of meat compared to flavor and color [44]. Muscle features, collagen, solubility, glycogen content, and the activities of proteases and their inhibitors are the most important physiological parameters that regulate meat tenderness [45]. Major variants in meat tenderness are connected to the variability in muscle attributes [46]. The high soluble collagen content in the longissimus thoracis muscle reflects prefer ability; this meat is the most tender and has less detectable connective tissue than other muscles [35]. In another study, Kadim et al. [10] found that infraspinatus, triceps brachii, and longissimus thoracis muscles in the camel had lower shear force values than semitendinosus, semimembranosus, and biceps femoris muscles, which might be because of less connective tissue. The longissimus thoracis muscle had the highest juiciness score and the semitendinosus and vastus lateralis muscles were less juicy than the psoas major, semimembranosus, and triceps brachii muscles.

## 5. Conclusions

In conclusion, there were significant differences between the chemical and structural characteristics of the LL, BF, and SM muscle samples among the three breeds of camel. Baladi Saudi and Pakistani had better meat quality traits than the Somali breed. In addition, LL muscles had better nutritional values and meat quality parameters than BF and SM muscles. Improvement in meat quality attributes were achieved with the aging process of 7 days. It is observed that, the Saudi Baladi camels have a merit of low fat content over both Somali and Pakistani camel breeds. This is in line with the national and international trends that encourage consuming low-fat food products for health reasons. It is also concluded that no significant effects were observed between the treatments as a result of storage period when sensory attributes were considered. Moreover, breed, muscle and storage period were interacted significantly only with regard to lightness color space and shear force. This is useful knowledge for the meat industry for optimizing processing and storage procedures for various camel muscles.

## Figures and Tables

**Table 1:** Effect of breed and muscle type on chemical composition of camel meat (n = 24/breed).

Treatments	Chemical Composition%			
	Moisture	Crude Protein	Crude Fat	Ash
**Effect of Breed (B)**				
Baladi Saudi	74.17	21.56	3.39 ^b^	0.88 ^b^
Pakistani	72.91	21.39	4.71 ^a^	0.99 ^a^
Somali	75.14	20.53	3.51 ^b^	0.82 ^b^
SE	0.42	0.39	0.20	0.04
*p*	NS	NS	<0.0001	0.01
**Effect of Muscle (M)**				
Longissimus lumborum	72.77	21.92 ^a^	4.37 ^a^	0.94 ^a^
Biceps femoris	75.10	20.45 ^b^	3.64 ^b^	0.81 ^b^
Semimembranosus	74.34	21.11 ^ab^	3.61 ^b^	0.94 ^a^
SE	0.43	0.39	0.20	0.04
*p*	NS	0.04	0.02	0.02
Interaction				
B × M	NS	0.04	NS	NS

^a,b,c^ Means within the same column with different superscripts are significantly different. SE, Standard error of mean; *p*, Probability level (significant at <0.05); NS, Not significantly different.

**Table 2:** Effect of breed, muscle type, and storage period and their interactions on pH and color components of camel meat.

Treatments	pH*_u_*	Color Coordinates			Chroma	b*/a* Ratio	Hue Angle
		L*	a*	b*			
**Effect of Breed (B)** (n = 24/breed)							
Baladi Saudi	5.87 ^b^	49.96 ^a^	14.05 ^b^	9.18	17.10	0.63	30.41
Pakistani	5.99 ^a^	49.92 ^a^	14.21 ^b^	8.36	16.79	0.59	28.81
Somali	5.90 ^b^	46.34 ^b^	15.88 ^a^	9.14	18.60	0.59	28.86
SE	0.03	0.83	0.55	0.46	0.64	0.04	1.37
*p*	0.01	0.01	0.03	NS	NS	NS	NS
**Effect of Muscle (M)** (n = 24/muscle)							
Longissimus lumborum	5.93	49.19	15.61 ^a^	9.77 ^a^	18.62 ^a^	0.66 ^a^	31.93 ^a^
Biceps femoris	5.94	49.13	13.99 ^b^	8.06 ^b^	16.46 ^b^	0.53 ^b^	26.72 ^b^
Semimembranosus	5.89	48.89	14.56 ^ab^	8.84 ^ab^	17.40 ^ab^	0.61 ^ab^	29.43 ^ab^
SE	0.03	0.83	0.55	0.46	0.64	0.04	1.37
*p*	NS	NS	0.01	0.03	0.04	0.04	0.03
**Effect of Storage period (S)** (n = 48/storage period)							
No storage	6.06 ^a^	48.56	13.47 ^b^	5.81 ^b^	14.96 ^b^	0.42 ^c^	21.51 ^b^
3 days storage period	5.83 ^b^	48.48	16.22 ^a^	10.64 ^a^	19.56 ^a^	0.64 ^b^	31.71 ^a^
7 days storage period	5.87 ^b^	49.18	14.46 ^b^	10.22 ^a^	17.96 ^a^	0.75 ^a^	34.86 ^a^
SE	0.03	0.83	0.55	0.46	0.64	0.04	1.37
*p*	<0.0001	NS	0.01	<0.0001	<0.0001	<0.0001	<0.0001
**Interactions**							
B × M	NS	NS	NS	NS	NS	NS	NS
B × S	0.01	0.01	0.02	<0.0001	0.01	0.02	0.01
M × S	0.02	NS	NS	NS	NS	NS	NS
B × M × S	NS	0.07	NS	NS	NS	NS	NS

^a,b,c^ Means within the same column with different superscripts are significantly different. SE, Standard error of mean; a* for redness, L* for lightness, and b* for yellowness; *p*, Probability level (significant at <0.05); NS, Not significantly different.

**Table 3:** Effect of breed, muscle type, and storage period on cooking loss (CL), water-holding capacity (WHC), myofibril fragmentation index (MFI), shear force (SF), and texture profile analysis (TPA) of camel meat.

Treatments	CL (%)	WHC Ratio	MFI Ratio	SF (N)	TPA			
					Hardness (N)	Springiness (cm)	Cohesiveness (−)	Chewiness(N × cm)
**Effect of Breed (B)** (n = 24/breed)								
Baladi Saudi	31.88 ^b^	0.39 ^ab^	41.79	29.71 ^b^	5.88	0.67	0.51	0.21
Pakistani	31.46 ^b^	0.41 ^a^	32.96	30.79 ^b^	5.10	0.65	0.51	0.21
Somali	34.28 ^a^	0.38 ^b^	39.75	46.58 ^a^	5.88	0.65	0.50	0.25
SE	0.70	0.01	3.02	0.12	0.04	0.01	0.01	0.03
*p*	0.01	0.01	NS	<0.0001	NS	NS	NS	NS
**Effect of Muscle (M)** (n = 24/muscle)								
Longissimus lumborum	29.87 ^b^	0.37 ^b^	40.09	31.28 ^c^	5.30	0.65	0.50 ^b^	0.24
Biceps femoris	33.66 ^a^	0.40 ^a^	33.56	40.31 ^a^	5.98	0.66	0.52 ^a^	0.23
Semimembranosus	34.09 ^a^	0.39 ^ab^	40.84	35.60 ^b^	5.69	0.65	0.50 ^b^	0.20
SE	0.70	0.01	3.02	0.12	0.04	0.01	0.01	0.03
*p*	<0.0001	0.03	NS	<0.0001	NS	NS	0.01	NS
**Effect of Storage period (S)** (n = 48/storage period)								
No storage	32.03	0.38	42.04 ^a^	36.68	6.08	0.65	0.50	0.26
3 days storage period	32.67	0.39	39.43 ^ab^	33.93	5.69	0.65	0.51	0.21
7 days storage period	32.92	0.40	33.01 ^b^	36.48	5.10	0.67	0.51	0.19
SE	0.70	0.01	3.02	0.12	0.04	0.01	0.01	0.03
*p*	NS	NS	0.01	NS	NS	NS	NS	NS
**Interactions**								
B × M	NS	NS	NS	NS	NS	NS	NS	NS
B × S	NS	NS	NS	0.01	NS	NS	NS	NS
M × S	NS	NS	NS	NS	NS	NS	NS	NS
B × M × S	NS	NS	NS	0.02	NS	NS	NS	NS

Cooling loss: LC; Water holding capacity: WCH; myofibril fragmentation index: MFI; Shear force: SF; and texture profile analysis: TPA. ^a,b,c^ Means within the same column with different superscripts are significantly different. SE, Standard error of mean; *p*, Probability level (significant at <0.05); NS, Not significantly different.

**Table 4:** Effect of breed, muscle type, and storage period on sensory evaluation attributes of camel meat.

Treatments	Sensory Evaluation Parameters				
	Color	Flavor	Tenderness	Juiciness	Acceptability
**Effect of Breed (B)** (n = 24/breed)					
Baladi Saudi	3.15 ^b^	3.65	3.98 ^a^	3.75 ^a^	3.81 ^a^
Pakistani	3.58 ^a^	3.64	3.94 ^a^	3.86 ^a^	3.86 ^a^
Somali	3.66 ^a^	3.55	3.39 ^b^	3.23 ^b^	3.48 ^b^
SE	0.11	0.08	0.09	0.08	0.08
*p*	0.01	NS	<0.0001	<0.0001	0.01
**Effect of Muscle (M)** (n = 24/muscle)					
Longissimus lumborum	3.20 ^b^	3.78 ^a^	4.09 ^a^	3.81 ^a^	3.93 ^a^
Biceps femoris	3.54 ^a^	3.47 ^b^	3.53 ^b^	3.50 ^b^	3.51 ^b^
Semimembranosus	3.65 ^a^	3.60 ^ab^	3.69 ^b^	3.53 ^b^	3.72 ^ab^
SE	0.11	0.08	0.09	0.08	0.08
*p*	0.01	0.03	<0.0001	0.01	0.01
**Effect of Storage period (S)** (n = 48/storage period)					
No storage	3.42	3.60	3.87	3.62	3.65
3 days storage period	3.63	3.67	3.74	3.55	3.80
7 days storage period	3.34	3.57	3.70	3.67	3.70
SE	0.11	0.08	0.09	0.08	0.08
*p*	NS	NS	NS	NS	NS
**Interactions**					
B × M	NS	NS	NS	NS	NS
B × S	NS	NS	NS	NS	NS
M × S	NS	NS	NS	NS	NS
B × M × S	NS	NS	NS	NS	NS

^a,b^ Means within the same column with different superscripts are significantly different. SE, Standard error of mean; *p*, Probability level (significant at <0.05); NS; Not significantly different.
